# Comparative Analysis of Complete Chloroplast Genomes and Phylogenetic Relationships in Medicinally Important Pantropical Genus *Bauhinia* s.s. (Leguminosae) from Southern Africa and Eastern Asia

**DOI:** 10.3390/ijms26010397

**Published:** 2025-01-05

**Authors:** Yanxiang Lin, Yuan Chen, Yanlin Zhao, Wei Wu, Chengzi Yang, Yanfang Zheng, Mingqing Huang

**Affiliations:** College of Pharmacy, Fujian University of Traditional Chinese Medicine, Fuzhou 350122, China; 2220408021@fjtcm.edu.cn (Y.C.); 2230408028@fjtcm.edu.cn (Y.Z.); 2240408016@fjtcm.edu.cn (W.W.); tiebaojin@163.com (C.Y.); yfzheng@fjtcm.edu.cn (Y.Z.)

**Keywords:** Cercidoideae, *Bauhinia*, chloroplast genome, positive selection, phylogenetic analysis, divergence time

## Abstract

*Bauhinia* s.s. belongs to the Cercidoideae subfamily, located at the base of the Leguminosae family. It displays a variety of growth habits and morphologies, and is widely utilized as both ornamental and medicinal plants globally. The objective of this research is to uncover chloroplast genomes of species from Eastern Asia and Southern Africa, thereby advancing our understanding of the diversity within this genus. This study sequenced *Bauhinia purpurea*, *Bauhinia brachycarpa* var. *microphylla*, *Bauhinia variegata* var. *candida*, *Bauhinia galpinii*, and *Bauhinia monandra* using the Illumina platform and conducted the construction of phylogenetic trees as well as the estimation of divergence times. Compared to Asian species, the IR regions of African species underwent a contraction of approximately 100–400 bp. The phylogenetic analysis indicated that Asian and African species clustered into two distinct clades, with high support. The divergence of *Bauhinia* s.s. species occurred in the late Paleocene, and the *rps18* and *cemA* genes were under positive selection. Six hypervariable regions were screened for evolutionary studies and the super-barcode data were used for species delimitation. The results revealed certain differences between African and Asian species in their chloroplast genomes of *Bauhinia* species.

## 1. Introduction

**Figure 1:**
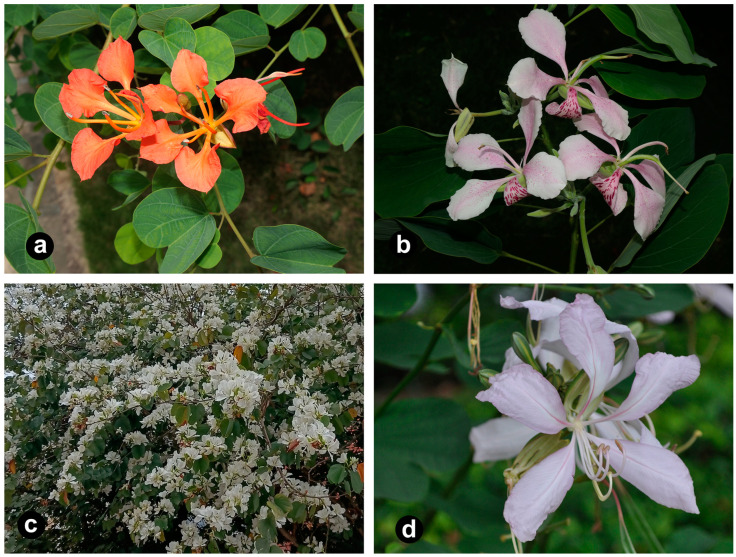
Photograph of floral morphology of *Bauhinia* s.s. species: (**a**) *Bauhinia galpinii*; (**b**) *Bauhinia monandra*; (**c**) *Bauhinia variegata* var. *candida*; (**d**) *Bauhinia purpurea*.

*Bauhinia* L. sensu lato (s.l.) belongs to the Cercidoideae subfamily of the Leguminosae family, with approximately 380 species [1], and has a pantropical distribution [2]. In honor of the Bauhin brothers, who made outstanding contributions to botany, Linnaeus named the genus *Bauhinia* L. [3]. Species of this genus are mostly trees or shrubs with bilobed leaves, and the calyx is spathaceous during anthesis, sometimes splitting into 2–5 lobes [4]. *Bauhinia* s.l. species, also known as the orchid tree, have distinctive leaf shapes, large and beautiful flowers (Figure 1), and boast a long florescence, such as *Bauhinia purpurea* and *Bauhinia galpinii*, as well as *Bauhinia* × *blakeana*, celebrated as the city flower of Hong Kong [5]. As an important horticultural group, these species are widely cultivated in gardens for ornamental purposes and as street trees, and they are loved by people worldwide.

*Bauhinia* s.l. is also recognized globally as an important medicinal genus [6,7,8]. For instance, *Bauhinia brachycarpa*, a traditional medicinal plant in China, offers its bark for treating diarrhea, tranquillizing, and alleviating pain, and modern pharmacological studies have demonstrated that its extracts possess analgesic properties [6,7]. The bark and roots of *Bauhinia variegata* serve to stanch bleeding and invigorate the spleen, while its leaves and flowers provide an antitussive effect [8]. The roots of *B. purpurea* function as a sedative-hypnotic drug among the Chinese Dong ethnic minority [9]. The acetone extract of *B. galpinii*, a traditional medicinal species in South Africa, exhibits potent antibacterial activity, aiding in diarrhea treatment [10,11]. In Brazil, the leaves of *Bauhinia monandra* are commonly used for diabetes treatment [12]. Additionally, a galactose-specific lectin from the leaves of *B. monandra* has anti-inflammatory and antinociceptive properties [13].

*Bauhinia* s.l. represents the most numerous and morphologically diverse group within Cercidoideae, with a global pan-tropical distribution, exhibiting varying habits and forms [1,14]. Traditional classification of *Bauhinia* s.l. relied on pollen characteristics, stamen morphology and number, and structure of mature anthers, which made it difficult to define species and genus relationships within this subfamily, leading to long-standing controversy of the delimitation in this genus [15,16,17,18,19,20]. For a period, *Bauhinia* L. was recognized as a broad concept encompassing around 300–380 species [17]. With the rapid development of molecular phylogenesis, molecular evidence has played an essential role in new taxonomic studies [21]. Recent phylogenetic research indicated that *Bauhinia* s.l. was polyphyletic [22,23,24,25]. In 2005, Lewis and Forest recognized it as eight genera, namely, *Bauhinia* L. s.s., *Piliostigma* Hochst., *Phanera* Lour., *Lasiobema* (Korth.) Miq., *Lysiphyllum* (Benth.) de Wit, *Barklya* F. Muell., *Tylosema* (Schweinf.) Torre & Hillc., and *Gigasiphon* Drake [2]. This view of subdividing *Bauhinia* s.l. into smaller genera gained support from Bruneau et al. [26], Hao et al. [27], and Sinou et al. [23], and was more widely accepted in subsequent research [1,14,24,28,29]. Since then, the boundaries of the genus underwent several taxonomic revisions. The latest phylogenetic analyses suggested that *Bauhinia* s.l. was divided into two major clades: the *Bauhinia* clade and the *Phanera* clade [1,30]. Unlike the *Phanera* clade, which consists of liana plants with tendrils, the *Bauhinia* clade is composed of *Bauhinia* s.s., *Brenierea* Humbert, and *Piliostigma*, which are non-tendril trees or shrubs. *Brenierea* is a monotypic genus endemic to Madagascar and is traditionally considered a sister genus to *Bauhinia* s.l. [2]. *Bauhinia* sensu stricto (s.s.) remains a large genus with about 230 species, distributed across Asia, Africa, and the Americas [1]. According to Sinou’s definition [1], the genus can be divided into eight sections, namely, sect. *Bauhinia*, sect. *Afrobauhinia*, sect. *Alvesia*, sect. *Amaria*, sect. *Micralvesia*, sect. *Pauletia*, sect. *Pseudophanera*, and sect. *Telestria*. Among them, sect. *Alvesia* and sect. *Micralvesia* are present in both Asia and Africa. Additionally, Asia also has sect. *Pseudophanera*, sect. *Pauletia* ser. *Acuminata*, and sect. *Telestria*, while Africa hosts sect. *Afrobauhinia*.

Fossils of *Bauhinia* s.l. plants have been widely discovered across various locations around the world [31]. The earliest known fossils originated from the Late Paleocene in China [32], along with fossils from the Late Eocene [33], Oligocene [22,31,34], Miocene [35,36], and Pliocene [36,37]; in Nepal, fossils ranging from the Miocene to the Pliocene have been found [38]; in India, numerous Miocene fossils have been found [38,39,40,41,42], as well as Pliocene fossils [43]; in Uganda, Pliocene fossils have been found [44]; and in Ecuador, Miocene fossils have been discovered [45]; These fossils exhibit a diversity of forms, such as leaves, pods, and wood. Palaeobiogeographic studies on the origin of *Bauhinia* s.l. are inconclusive, with some suggesting potential origins in the Paleocene Laurasia [34], the low-latitude regions of the Eastern Tethys seaway [31,35], or possibly in the Afrotropical realm, following an “out of Africa” model [32]. Nonetheless, *Bauhinia* s.l. has undergone rapid evolutionary radiation, leading to its current species distribution pattern. Consequently, this cosmopolitan genus provides excellent material for the studies on adaptive evolution in the context of long geological history.

An increasing number of studies have demonstrated that genomic data serve as a crucial resource for research in plant genetics and breeding, biological evolution, phylogeny, and related fields [46,47,48,49]. The chloroplast (cp) genome is one of the primary locations for cytoplasmic genetic material in green plant cells, and all genes present within the cp are collectively referred to as the cp genome [50]. Typically, the cp genome is a circular double-stranded molecule, ranging in size from 120 to 200 kb, with two inverted repeat (IR) regions separated by a large single-copy (LSC) region and a small single-copy (SSC) region that are non-repetitive. This genome is maternally inherited and possesses a relatively independent genome and genetic sequence [50]. Chloroplasts in plant cells, as semi-autonomous replicating organelles, have a genome that, compared to the nuclear and mitochondrial genomes, is not only structurally simpler but also typically contains several thousand copies, making it easy to obtain a complete cp genome sequence. Therefore, it holds vital value in revealing origins of species, evolutionary processes, and the phylogenetic relationships among different species [51,52,53]. Due to its complex morphology and challenging identification, the development of molecular markers is highly necessary for *Bauhinia* s.s.

The current research focuses on the taxonomy and biogeography of this genus, with numerous gene fragments from Cercidoideae reported [1,14,23,25,54]. However, there are relatively few reports on the complete cp genomes for this group, especially for *Bauhinia* s.s., and even less comprehensive comparative analyses of the cp genomes. This hinders our understanding of the genetic characteristics and evolutionary history of this taxon. In the process of adaptive evolution, natural selection serves as a significant driving force for evolution [55]. An intriguing question is whether there are differences in the cp genomes of *Bauhinia* s.s. species from Asia and Africa during this process. Therefore, this study collected three species distributed in Asia, namely, *B. purpurea*, *Bauhinia brachycarpa* var. *microphylla*, and *Bauhinia variegata* var. *candida*, as well as *B. galpinii* from southern Africa and *B. monandra* from Madagascar. We sequenced and assembled the complete cp genomes of these species, and conducted comparative analyses and phylogenetic tree construction. Moreover, these species all possess medicinal value, and we aim to delineate species within *Bauhinia* s.s. using super-barcode data from the entire chloroplast to assist in the identification of these medicinal plants.

## 2. Results

### 2.1. Chloroplast Genome Characteristics of Bauhinia s.s.

**Figure 2:**
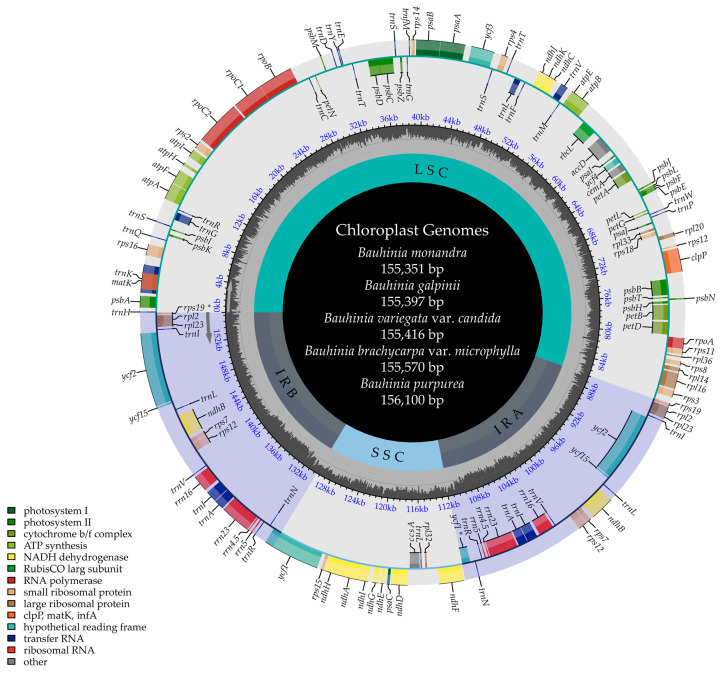
Chloroplast genome maps of five newly sequenced *Bauhinia* s.s. species. The outermost circle depicts gene direction, with the innermost circle illustrating the LSC/SSC/IR regions. Genes from various functional groups are represented by different colors.

In this study, five *Bauhinia* s.s. species were sequenced and assembled, with the cp genomes ranging from 155,351 to 156,100 bp. These genomes exhibit a typical quadripartite structure, comprising a large single-copy (LSC) region (86,111–86,688 bp), a small single-copy (SSC) region (17,922–18,499 bp), and a pair of identical inverted repeat (IR) regions (25,105–25,847 bp) (Figure 2). The overall GC content of the cp genomes ranged from 36.24% to 36.46%, with the GC content in the IR regions (42.31–42.54%) being higher than that in the LSC regions (33.90–34.19%) and SSC regions (30.15–30.52%) (Table S1). There was no significant difference in the overall GC content between the African species *B. galpinii* and *B. monandra* and the three Asian species.

The cp genomes of the five *Bauhinia* species contained a total of 128 genes, including 83 protein-coding genes (CDS), 37 tRNA genes, and 8 rRNA genes (Table S2). These genes can be classified into four categories: 45 genes related to photosynthesis, 73 genes involved in self-replication, 5 other genes, and 5 genes with unknown functions. Additionally, among the 128 genes, 17 genes contained one intron, including 9 CDS (*ndhA*, *ndhB* × 2, *petB*, *petD*, *atpF*, *rpl16*, *rps16*, *rpoC1*) and 8 tRNA genes (*trnA*-*UGC* × 2, *trnG*-*UCC*, *trnI*-*GAU* × 2, *trnK*-*UUU*, *trnL*-*UAA*, *trnV*-*UAC*), while 4 genes (*rps12* × 2, *clpP*, *ycf3*) contained two introns.

### 2.2. Structural Variation in the Chloroplast Genomes

The boundaries of the cp genomes in *Bauhinia* species were relatively conserved (Figure S1). The IR regions of *B. brachycarpa* var. *microphylla* and *B. variegata* var. *candida*, which were distributed in Asia, were similar in length. However, *Bauhinia variegata* var. *candida*, distributed in South Asia, had an IR region that was approximately 300 bp longer compared to these two species. Conversely, *B. monandra*, found in Madagascar, and *B. galpinii* from southern Africa showed contracted IR regions, with the former being nearly 150 bp shorter and the latter being nearly 500 bp shorter than the Asian species. Regarding the composition and distribution of boundary genes, the boundary genes of the five species were all located at the *ycf1* and *rps19* genes, with only minor positional differences observed, and no loss of the inverted repeat regions was detected. The boundaries between LSC and IRb were conserved, with all species having their boundary located within the coding region of the *rps19* gene. At the boundary between IRb and SSC, the *ndhF* gene was located 156 and 162 bp downstream of the boundary in *B. brachycarpa* var. *microphylla* and *B. monandra*, respectively, while the *ndhF* genes in the other three species were pseudogenized. The boundaries between SSC and IRa were located within the coding region of the ycf1 gene, with the start codon of *B. purpurea* shifted towards the IRa region by 1000 bp, while the other species showed shifts of 760 to 800 bp. The boundary between IRa and LSC was highly conserved, being located 12 to 15 bp upstream of the *trnH* gene in all species. Additionally, no rearrangements or inversions were observed in these genomes.

### 2.3. Codon Usage Patterns in the Chloroplast Genomes

**Figure 3:**
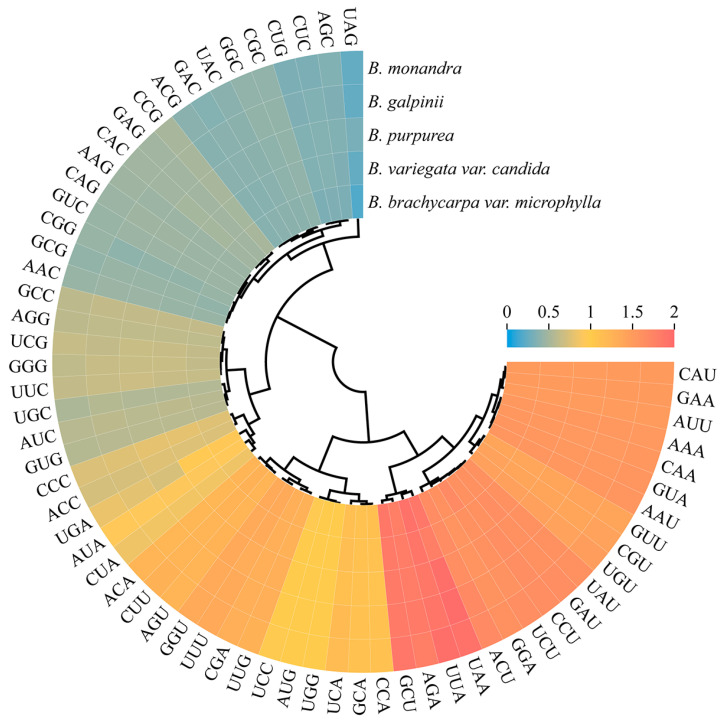
The relative synonymous codon usage (RSCU) values of each codon in the chloroplast genome of *Bauhinia* s.s. species. Red represents a high RSCU value, indicating that the codons have a preference; blue represents a low RSCU value, indicating a non-preferred codon; yellow represents an RSCU value of 1, indicating no preference for codons.

An analysis of the relative synonymous codon usage (RSCU) preference was conducted for *B. brachycarpa* var. *microphylla*, *B. variegata* var. *candida*, *B. purpurea*, *B. galpinii*, and *B. monandra*, with the RSCU values calculated for 61 codons. Among these five species, 29 codons showed a preference (RSCU > 1), with high RSCU values (RSCU > 1.60) including UUA encoding leucine, GCU encoding alanine, AGA encoding arginine, ACU encoding threonine, UCU encoding serine, CCU encoding proline, GAU encoding aspartic acid, and UAU encoding tyrosine (Figure 3, Table S3). The UUA codon encoding leucine had the highest RSCU values (1.87–1.94), indicating that this codon was preferred in *Bauhinia* s.s. Conversely, the CUG codon encoding leucine had the lowest RSCU values, suggesting it as a non-preferred codon in this genus. Among all amino acids, leucine was the most common, accounting for 10.27–10.44% of all codons, while cysteine was the least frequent, constituting only 1.08–1.01% of codons. In terms of individual codon counts, AUU encoding isoleucine was the most abundant, with 846–905 occurrences in the genome, while UGC encoding cysteine was the least frequent, occurring only 59–62 times.

**Figure 4:**
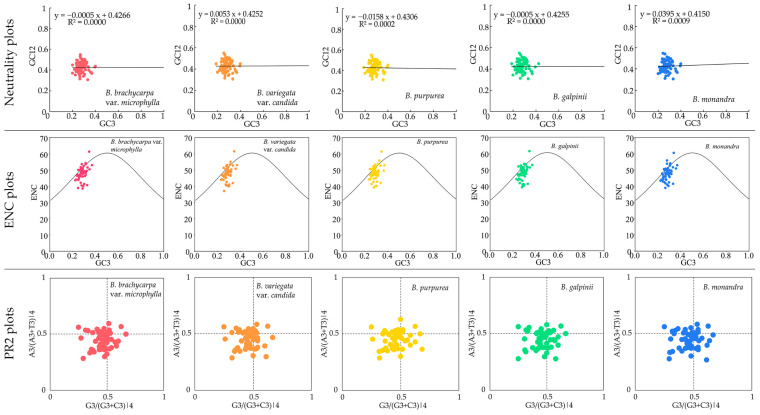
The codon usage bias analysis of *Bauhinia* s.s., including the neutrality plots, Effective Number of Codons (ENC), and Parity Rule 2 (PR2). The various colored dots in the figure denote protein-coding genes from different species. A regression coefficient (R^2^) in the neutrality plots close to 1, genes in the ENC Plots aligning with or residing on the standard curve, and a PR2 scatter plot falling at the center point, all suggest that mutational pressure is the primary determinant of codon preference. Conversely, an R^2^ nearing 0, genes significantly deviating from the standard curve in ENC plots, or the genes straying from the center in PR2 plots, indicate that natural selection plays a dominant role.

Neutrality plots were used to assess the relationship between natural selection and mutational pressure by analyzing the correlation between GC12 (the average of GC1 and GC2) and GC3. The results indicated that *Bauhinia* s.s. had GC3 values ranging from 18.75 to 42.50% and GC12 values from 30.77 to 54.88%, with regression coefficients of −0.0158 to 0.0395 and determination coefficients of 0 to 0.0009 (Figure 4, Table S4). Therefore, the non-significant correlation between GC12 and GC3 suggested that codon preference in *Bauhinia* s.s. cp genomes was more influenced by natural selection than by base mutations. In the Effective Number of Codons (ENC) analysis, most genes deviated from the standard curve and fell below it, with the ENC values all above 36 and a small number distributed along the standard curve (Table S5). This indicated that codon preference in *Bauhinia* s.s. is weak and primarily influenced by natural selection. In the Parity Rule 2 (PR2) plot analysis, the distribution of all genes on the plot across the four quadrants was uneven, with most genes located in the quadrant where G3/(G3 + C3)|4 < 0.5 and A3/(A3 + T3)|4 < 0.5 (Table S6). The results showed that the usage frequency of the third base was C > G and T > A, further confirming that codon preference in *Bauhinia* s.s. cp genomes was mainly influenced by natural selection.

### 2.4. Dispersed and Simple Sequence Repeat Identification

**Figure 5:**
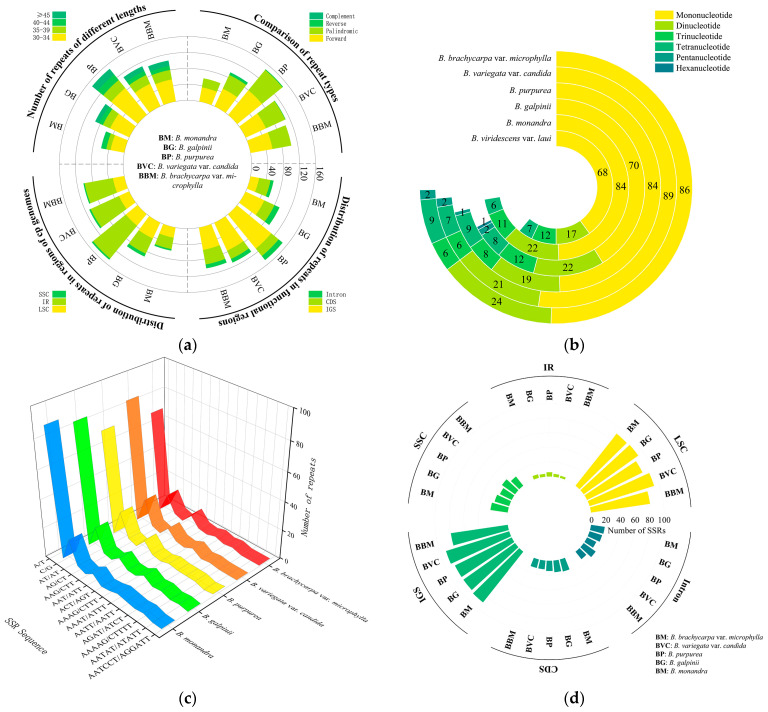
Distribution of quantities among various types of repeats. (**a**) The quantities are depicted as follows: the top-left quadrant categorizes sequences by length, the top-right quadrant identifies four types of repeats, the bottom-left quadrant delineates quadrant structural partitions, and the bottom-right quadrant indicates the counts within CDS, IGS, and introns; (**b**) the number of six types of simple sequence repeats (SSRs); (**c**) the number of SSRs with different motifs; (**d**) the number of SSRs in different regions.

We identified and statistically analyzed the repetitive sequences in the cp genome sequences of *Bauhinia* s.s., and the results revealed that among the four types of dispersed repeats, forward repeats (50.91–61.02%) and palindromic repeats (38.89–48.18%) accounted for the largest proportions (Figure 5a, Table S7). Inverted repeats followed with a smaller proportion (0–6.38%), and complementary repeats were the least frequent (0–1.06%). All dispersed repeats were longer than 30 bp. Among the dispersed repeats of all lengths, the largest proportion was found in the 30–39 bp range (75.68–78.18%). Except for *B. monandra* (37.29%), these dispersed repeats were primarily distributed in the IGS regions (54.81–75.00%) (Table S8). Interestingly, the proportion of dispersed repeats in the IR regions of *B. monandra* and *B. galpinii*, which were distributed in Africa, was relatively smaller (44.68–49.15%) compared to the Asian species (69.23–71.62%).

We counted a total of 104–125 simple sequence repeats (SSRs) in the cp genomes of the five *Bauhinia* s.s. species (Figure 5b, Table S9). Six types of SSR were identified: mononucleotide, dinucleotide, trinucleotide, tetranucleotide, pentanucleotide, and hexanucleotide repeats. Mononucleotide repeats comprised the largest proportion (60.87–71.20%), with A/T motif mononucleotide repeats (60.87–70.40%) far outnumbering other types (Figure 5c). Pentanucleotide and hexanucleotide repeats were found in African species and *B. purpurea* distributed in South Asia, but were not observed in other Asian species (Table S10). In the quadripartite structure, these SSRs were predominantly distributed in the LSC regions (72.80–78.85%), with the largest proportion of SSRs found in the IGS regions (71.20–75.96%) (Figure 5d).

### 2.5. Screening of Hypervariable Regions and Adaptive Evolution Analysis

**Figure 6:**
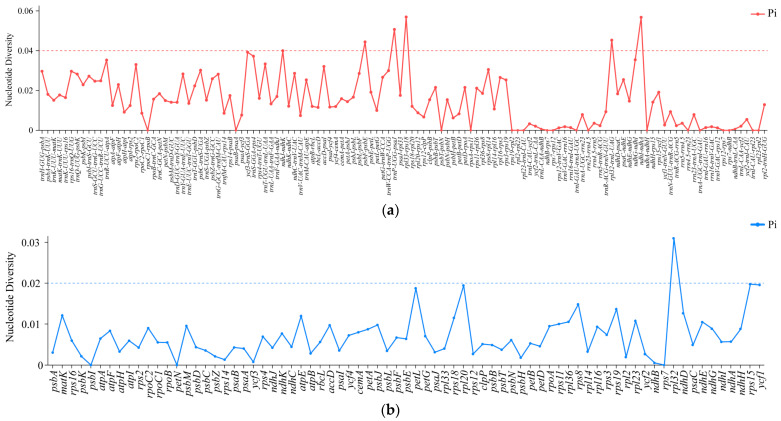
Analysis of nucleotide polymorphism (Pi) in *Bauhinia* s.s. (**a**) The Pi values of the intergenic spacers genes; (**b**) the Pi values of the protein-coding genes.

We conducted a nucleotide polymorphism (Pi) analysis on the cp genomes of *Bauhinia* s.s. species. The results indicated that six IGS regions had high Pi values (Pi ≥ 0.04), including *rpl33*-*rps18*, *ndhI*-*ndhA*, *trnP*-*psaJ*, *rpl32*-*trnL*, *psbF*-*psbE*, and *ndhJ*-*ndhK* (Figure 6, Table S11). Among these, *rpl33*-*rps18*, *trnP*-*psaJ*, *psbF*-*psbE*, and *ndhJ*-*ndhK* were located in the LSC region, while *ndhI*-*ndhA* and *rpl32*-*trnL* were situated in the SSC region. We also detected structural variations in the cp genomes of *Bauhinia* s.s. and found that the LSC region had a higher degree of variation than that in the SSC region, with the IR region showing the lowest variation (Figure S2).

**Figure 7:**
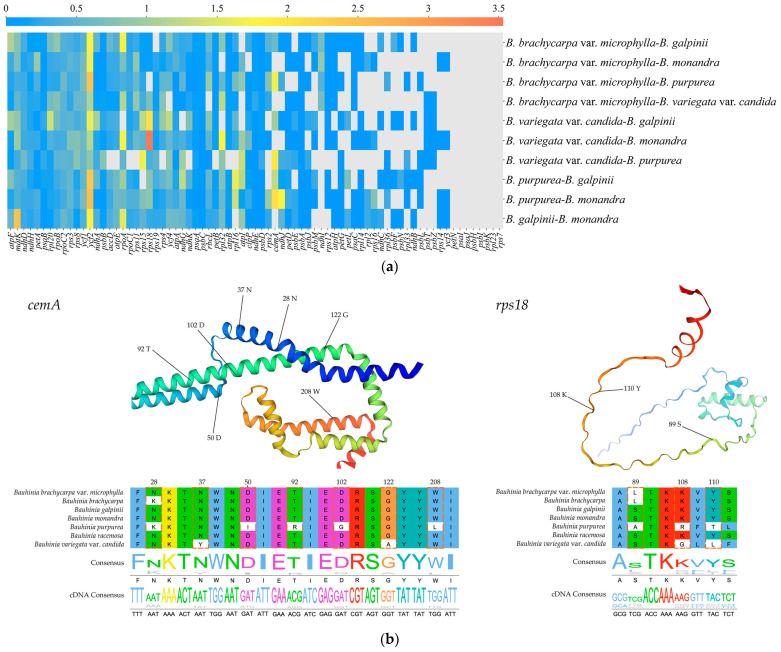
Analysis of selective pressure in the chloroplast of *Bauhinia* s.s. (**a**) The heatmap shows the paired Ka/Ks ratio of each individual gene. The values within the squares represent the Ka/Ks ratio, where red and yellow (Ka/Ks > 1) indicate positive selection, and blue (Ka/Ks < 1) indicates purification selection. (**b**) Amino acid sequence and spatial distribution of positive selection sites within the *cemA* and *rps18* genes. The red box indicates the loci with *p*-value < 0.05 and Bayesian empirical Bayes posterior probability > 0.95.

Selective pressure analysis was performed to detect the selective pressures acting on genes in *Bauhinia* s.s. species. Genes with Ka/Ks ≥ 45 or NA were excluded from the analysis due to the near absence of nonsynonymous sites. Our results showed that most genes were under purifying selection (Ka/Ks < 1), while 15 genes may be undergoing positive selection (Ka/Ks > 1), including *rps18*, *ycf2*, *matK*, *cemA*, *rpoA*, *ndhJ*, *rpl32*, *rps15*, *rpl16*, *rpl20*, *atpI*, *ndhG*, *ycf4*, *atpF*, and *ndhA* (Figure 7a). Branch-site analysis further revealed that among the 69 CDS, the *p*-values for the *cemA* gene (3.39 × 10^−4^) and the *rps18* gene (4.73 × 10^−7^) were both less than 0.05 (Figure 7b), and the BEB values for codons at the 28th (0.997), 37th (0.970), 50th (0.997), 92nd (0.970), 102nd (0.970), 122nd (0.970), and 208th (0.970) positions of the *cemA* gene, and the 89th (0.973), 108th (0.995), and 110th (1.0) codons of the *rps18* gene, were all greater than 0.95. Therefore, these sites were considered to be under positive selection. The positively selected sites in the *cemA* gene were all located within the α-helix of the protein, while the 89th and 108th sites of the *rps18* gene were also located in the α-helix, and the 110th site was situated in a coil region.

### 2.6. Biogeographic Evolution and Species Delimitation

**Figure 8:**
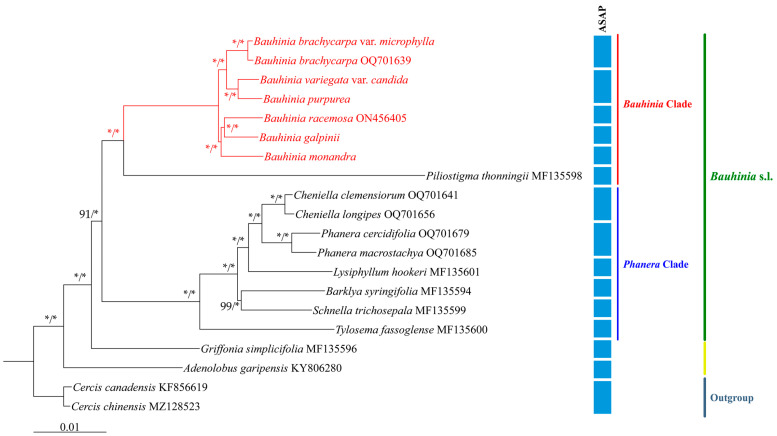
Maximum likelihood (ML) and Bayesian inference (BI) phylogenetic trees for 20 species of Cercidoideae, reconstructed using the complete chloroplast genomes. The support values on the branch are displayed in the order of BP_ML_/PP_BI_. “*” indicates that the support value BP = 100 or PP = 1.0. The blue squares represent the results of the molecular species delimitation analysis.

To elucidate the phylogenetic relationships among *Bauhinia* s.s. species, this study reconstructed the phylogeny of *Bauhinia* s.s. based on the complete cp genomes using ML and BI methods. The results indicated a high degree of consistency between the two methods, with all branches receiving strong support (Figure 8). With *Cercis* species as the outgroup, *Adenolobus* and *Griffonia* represented the basal taxa within Cercidoideae, forming a sister-group relationship with *Bauhinia* s.l. *Bauhinia* s.l. was further divided into two strongly supported clades: the clades of *Bauhinia* and *Phanera*. Within the *Bauhinia* clade, *Piliostigma* species occupied a basal position and clustered together with other species of *Bauhinia* s.s., with strong support from the data. Among *Bauhinia* s.s., each subclade received strong support. Specifically, *Bauhinia racemosa* clustered with *B. galpinii*, and this clade was then associated with *B. monandra*, comprising a group of African and South Asian species. In another branch, *B. variegata* var. *candida* grouped with *B. purpurea*, while *B. brachycarpa* var. *microphylla* and *Bauhinia brachycarpa* formed another group. These species together formed a group of Asian species. Furthermore, the Automatic Partitioning (ASAP) molecular delimitation method was applied to chloroplast genome datasets of seven *Bauhinia* s.s. for species delimitation (molecular operational taxonomic units, MOTUs). The results indicated that these datasets were delineated into five MOTUs. *B. brachycarpa* and *B. brachycarpa* var. *microphylla* were delineated into the same MOTU, while *B. purpurea* and *B. variegata* var. *candida* were delineated as the same MOTU. The remaining species were each identified as individual MOTUs. In these MOTUs, we detected a total of 626 to 2083 the molecular diagnostic characteristics (MDC) of chloroplast genomes. The number of MDCs found in MOTU-1 and MOTU-2 was the least, while the number of MDCs found in MOTU-3 and MOTU-4 was the most (Table S12).

**Figure 9:**
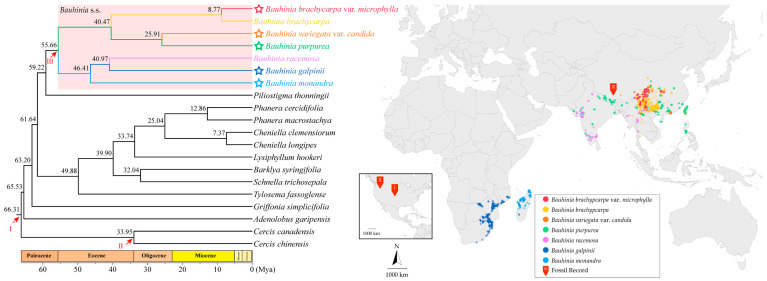
Left figure shows the estimated divergence time of *Bauhinia* s.s. taxa, with the numbers next to the nodes indicating the divergence time (Mya, million years ago). The arrows indicate fossil calibrations, corresponding to the fossil records depicted on the accompanying map to the right via Roman numerals. The right figure shows the world distribution map of the living species for *Bauhinia* s.s. included in this study. Data of specimens are sourced from the Global Biodiversity Information Facility website (https://www.gbif.org/ (accessed on 20 November 2024)).

The phylogenetic analysis of divergence times reconstructed the overall divergence timeline for *Bauhinia* s.l. (Figure 9). The divergence time between *Bauhinia* s.l. and its basal lineage was in the Paleocene period (ca. 63.20 Mya, 95% Highest Posterior Density (HPD): 60.80–65.43 Mya). Within *Bauhinia* s.l., the *Bauhinia* clade diverged from the *Phanera* clade around 61.64 Mya (95% HPD: 59.04–64.07 Mya), and *Bauhinia* s.s. diverged around 59.22 Mya (95% HPD: 56.79–61.77 Mya). The Asian and African lineages diverged in the early Eocene (ca. 55.66 Mya, 95% HPD: 54.90–56.79 Mya).

## 3. Discussion

### 3.1. Basic Characteristics of the Chloroplast Genomes

*Bauhinia* s.l. belongs to the Cercidoideae subfamily of the Leguminosae family and possesses high ornamental and medicinal value. The *Bauhinia* genus has a pantropical distribution and is utilized worldwide as horticultural flowers and medicinal plants [33]. Previous studies based on cp genome DNA fragments (*matK*, *trnL*-*trnF*) have greatly enhanced our understanding of the phylogeny of *Bauhinia* s.l. [1,14]. Comparative analyses of the cp genomes among the genera of Cercidoideae revealed structural variations such as intron losses, inversions, and shifts of IR boundaries, demonstrating that the evolution of the cp genome in early-branching lineages of legume plants has not stagnated [23,56]. However, most of the research to date focused on systematic taxonomy, with fewer comparative analyses of the cp genome, especially for *Bauhinia* s.s. species. *Bauhinia* s.s. is widely distributed in Asia, Africa, and the Americas, with current research suggesting that this group originated in Africa or Asia.

In this study, we sequenced and assembled the cp genomes of five *Bauhinia* s.s. species distributed in Africa and Asia, elucidating their characteristics and genetic relationships, and further supplemented the comparative genomic analysis of *Bauhinia* s.s. The structures and lengths of the five cp genomes were consistent with those previously reported for the cp genomes of *Phanera* plants [57], with minor variations among different genomes, and the structures and gene sequences are highly conserved. These cp genomes ranged in size from 155,351 bp (*B. monandra*) to 156,100 bp (*B. purpurea*) and are composed of the typical quadripartite structure, including the LSC, SSC, and IR regions. The expansion and contraction of genome boundaries are manifestations of the evolutionary process [58]. The distribution of gene types at the IR boundaries in *Bauhinia* s.s. was similar, and the overall gene structure and arrangement order were generally similar, indicating that the IR boundaries were relatively stable. However, there was still some variation in the IR boundaries among the genomes of *Bauhinia* s.s. species. Compared to other Asian species, the IR region of *B. purpurea*, distributed in South Asia, showed an expansion of about 300 bp, while the IR region of African species (25,105–25,401 bp) was contracted by 100–400 bp. This contraction and expansion of the IR region may be related to the double-strand break (DSB) model [56,59] and illegitimate recombination [60,61].

Interestingly, in the cp genome structure, the GC content (ranging from 42.31% to 42.54%) in the IR regions was higher than that in other regions, potentially correlated with the exclusive location of rRNA within the IR regions. There was a subtle difference in the total GC content between African and Asian species, with African species having a slightly lower GC content than Asian species. This could lead to accelerated cp gene mutations and alterations in gene expression, the phenomenon commonly observed in most green plants [62]. Additionally, the *rpl2* gene in the five cp genomes lost its intron, a phenomenon also reported in some legume plants [23,56,63,64], thereby validating the studies by Wang et al. [56] and Lai et al. [64]. No inversions or rearrangements similar to those observed in the *Tylosema* genus were detected in *Bauhinia* s.s., suggesting a relatively conserved cp genome structure. It is noteworthy that expanding the sampling of *Bauhinia* s.s. species in Africa and Asia will contribute to further assessing and validating the variations within their chloroplast genomes.

### 3.2. Repeat Sequences and Nucleotide Polymorphism

Repeat sequences are widely distributed in the cp genomes of plants, serving as an important basis for studying species genetic characteristics and biological evolution [53]. SSR sequences are associated with gene expression, transcription, and protein function. Due to various factors such as selective pressure and gene mutations, the distribution and types of SSRs may vary greatly among different species. In this study, a total of 104–125 SSR loci were identified across five cp genomes, with the abundance of SSRs distributed in the LSC region, potentially related to the functional diversity of genes in this partition. *Bauhinia* s.s. species had a high content of A/T motifs, which may be associated with the relatively easier breakage of A/T bases compared to G/C bases during gene recombination [65]. This study indicated that pentanucleotides were present in African species and *B. purpurea* distributed in South Asia, while hexanucleotides were only found in *B. purpurea*, with the other two Asian species lacking these types of repeated SSRs. These SSRs may be applicable for further species identification.

We also observed differences in the dispersed repeats of the cp genomes of *Bauhinia* s.s. distributed in Africa and Asia. The proportion of dispersed repeats in the IR regions of *B. monandra* and *B. galpinii*, distributed in Africa (44.68–49.15%), was relatively smaller compared to the Asian species (69.23–71.62%). This difference may be due to African species maintaining higher genomic stability to adapt to their growth environments. The dispersed repeats of the five species were mainly forward and palindromic repeats, and the number of such repeats was smaller in African species than Asian species, which may be a result of the combined effects of geographical factors and genetic variation.

We identified numerous variation sites in the non-coding regions of cp genomes, which may be suitable for development as nucleotide polymorphism sites for interspecific genetic diversity analysis [66,67,68]. In this study, six IGS regions with high Pi values (Pi ≥ 0.04) were screened, namely, *rpl33*-*rps18*, *trnP*-*psaJ*, *psbF*-*psbE*, and *ndhJ*-*ndhK* located in the LSC region, and *ndhI*-*ndhA* and *rpl32*-*trnL* situated in the SSC region. These sites may be further utilized in the studies of gene polymorphism.

### 3.3. Phylogenetic Relationships, Species Delimitation, and Divergence Times

The high conservation and maternal inheritance of the cp genomes provide new insights into plant phylogenetic studies [50]. In this study, under the new taxonomic system of Leguminosae [14], chloroplast genomes were utilized to clarify the phylogenetic relationships of *Bauhinia* s.s., with representative species from Asia and Africa included. Species within the Cercidoideae subfamily exhibited complex trait variations, and previous studies based on genome fragments showed low support for the two major clades of *Bauhinia* s.l. [1,23,69], with phylogenetic relationships remaining unresolved. The phylogenetic analysis based on the cp genomes of *Bauhinia* s.s. in this study revealed high support for the two lineages of *Bauhinia* s.l., consistent with our previous findings [57]. Within the *Bauhinia* clade, *Bauhinia* s.s. was most closely related to *Piliostigma*. In the phylogenetic tree constructed by Sinou [1] based on *Legcyc1*, *Legcyc2*, *matK*, and *trnL*-*F*, *B. galpinii* belonged to the sect. *Afrobauhinia* clade, while *B. monandra* belonged to another sect. *Afrobauhinia* clade, separated by the Asian clade. However, in our study, the African species *B. monandra* clustered with *B. galpinii*, receiving good support. To better resolve the intergeneric relationships within *Bauhinia* s.l. and test the robustness of the phylogenetic relationships between the two lineages of *Bauhinia* s.l., it is still necessary to expand the sampling of this group to enhance the reliability of the results.

Currently, DNA barcoding has been widely applied in species identification. However, for morphologically highly similar and taxonomically complex groups, the results of DNA barcoding identification are sometimes difficult to verify morphologically [70], which may be due to taxonomic over-splitting in morphology or differing opinions on the same morphological characteristics [71,72]. The molecular species delimitation results generally align with morphological classifications, providing great assistance in validating the taxonomic status of known species and enhancing species identification efficiency. Therefore, it is necessary to redefine species based on molecular methods [73]. Some cases have shown that using the complete cp genome sequence as a DNA super barcode provides stronger discriminating power [74]. For instance, in the research of Hu et al. [75], the DNA super barcode of *Roscoea* species showed the expected identification results (90%); the super barcode also achieved a high success rate (91.67%) in identifying *Fritillaria taipaiensis* and its related species [76], as well as a high success rate (95.54%) in distinguishing *Calypogeia* species [77]. *Bauhinia* s.s. is a complex taxonomic group with significant medicinal and ornamental value. The effective barcodes for species identification within this group are still lacking. This study preliminarily explored the identification of medicinal plants within this group using the complete cp genomes as super barcodes. The results indicated that out of seven *Bauhinia* s.s. plants, five were identified. Although not reaching the success rates reported for super barcoding mentioned previously, the species delimitation success rate for this group remained relatively high (71.43%). *B. brachycarpa* var. *microphylla*, a variety of *B. brachycalpa*, was identified within the same group, which was also supported by morphological evidence. Despite the recognized morphological differences between *B. purpurea* and *B. variegata* var. *candida*, ASAP classified them as the same species, and their relationship requires further investigation. Given the limited number of species involved in this study, the current results are insufficient to demonstrate the species identification capability of super barcodes in *Bauhinia* s.s. Further sampling should be expanded to obtain more convincing research outcomes.

Based on the distribution of extant species of *Bauhinia* s.s., African species and Asian species clustered into two distinct clades, with species from the Indian Plate (South Asia) scattered among these two clades, forming a bridge connecting the “Asian-African” species. The phylogenetic relationships between Asian and African species were, to some extent, reflected in their geographical distribution, suggesting that regardless of whether the group originated in Africa [2,32] or eastern Tethys Seaway (Asia) [31,34,35,37], the species dispersal of this group passed through the Indian Plate and Madagascar. Molecular clock estimations indicated that the most recent common ancestor of *Bauhinia* s.s. originated in the late Paleocene (ca. 59.22 Mya, 95% HPD: 56.79–61.77 Mya), while the Asian and African clades diverged during the early Eocene (55.66 Mya, 95% HPD: 54.90–56.79 Mya). The formation of the monsoon-controlled climate pattern may be a vital factor driving the migration of *Bauhinia* s.s. to new regions. Temperature change in precipitation patterns may also alter ecosystems, directly impacting the habitats of *Bauhinia* s.s. species. Further research on the historical biogeography of *Bauhinia* s.s. still requires more fossil evidence and molecular data.

### 3.4. Adaptive Evolution in Bauhinia s.s.

Factors influencing codon usage bias include natural selection and genetic mutation [78]. GC content can also be used for genome identification and differentiation among various genomes. Analysis of genome codon usage bias and related factors can be employed for gene function prediction. In this study, the cp genomes of the five *Bauhinia* s.s. plants exhibited similar codon usage patterns, with similar analytical results observed between species distributed in Asia and those in Africa. Results from neutrality analysis, ENC analysis, and PR2 bias analysis indicated that codon preference in *Bauhinia* s.s. was more influenced by natural selection than by base mutations.

Selective pressure analysis identified fifteen genes under positive selection. We hypothesize that these genes adapt to different ecological environments in Africa and Asia by influencing photosynthesis, gene expression, and transcription. Specifically, the genes *atpF*, *atpI*, *ndhA*, *ndhG*, and *ndhJ* are crucial in plant photosynthesis. The *atpF* gene encodes the b-subunit of the F0 sector of ATP synthase and is co-transcribed with the *atpI* gene [79]. ATP synthase is a key enzyme in energy metabolism [80], and the adaptive evolution of the *atpF* and *atpI* genes may impact energy metabolism of chloroplasts. The *ndhA*, *ndhG*, and *ndhJ* genes are membrane subunits involved in encoding NADH dehydrogenase located in the thylakoid membrane. Their adaptive evolution may affect energy conversion and alleviate oxidative stress [81]. The genes *rpl16*, *rpl20*, *rpl32*, *rpoA*, *rps15*, and *rps18* participate in gene expression and transcription processes related to plant growth and development. Among them, *rpl16*, *rpl20*, and *rpl32* play important roles in ribosome synthesis and function, further influencing the cell growth rate. The *rpo* genes encode the α-subunit of RNA polymerase, thereby affecting transcription processes in chloroplasts. The *rps* gene family catalyzes protein synthesis and maintains cell growth. The genes *cemA*, *matK*, *ycf2*, and *ycf4* indirectly influence photosynthesis and growth and development processes. Specifically, *matK* encodes a maturase associated with intron splicing in chloroplast group II introns, affecting the transcription and processing of chloroplast genes. The *ycf4* gene influences the accumulation of photosystem I [82]. The evolutionary significance of *ycf2* remains unclear and awaits further elucidation of its gene function.

Focusing on the Asian clade as the foreground branch, we found that the *rps18* and *cemA* genes were under positive selection. Recent studies showed that the *rps18* gene played a vital role in the growth and development of leaves and was crucial for cell survival [83,84]. We found that, compared to the *rps18* gene in the African species, the 89th, 108th, and 110th sites of the *rps18* gene in the Asian species were under positive selection. We also observed that the leaf traits of the African species *B. galpinii* and *B. monandra* were characterized by an obtuse apex and a cuneate to shallow cordate base, while the leaves of the three Asian species tend to have a deeper cordate base and narrower leaf width. We speculated that after the divergence of *Bauhinia* s.s. species in the late Paleocene, their leaves underwent specific adaptive evolution in photosynthesis and cp function to adapt to different growth environments of Asia and Africa. This may also be related to protein synthesis, which may be upregulated or downregulated under certain conditions to help cells adapt to environmental changes. Additionally, the *cemA* gene was under positive selection. This gene encodes the cp envelope membrane protein, and disruption of the gene will increase photosensitivity and affect CO2-dependent photosynthesis and inorganic carbon absorption [85]. The positive selection of this gene may be related to the adaptation of *Bauhinia* s.s. species to solar radiation in Asia. The cp genomic data available for these species are currently limited, and we believe that it is necessary to expand the sampling to assess the impact of positive selection on this gene in *Bauhinia* s.s. plants and to explore the causes of positive selection at these sites.

## 4. Materials and Methods

### 4.1. Plant Material, DNA Extraction, and Sequencing

Twenty cp genome sequences were utilized, with fifteen of these sequences downloaded from NCBI (https://www.ncbi.nlm.nih.gov (accessed on 20 November 2024)), and the remaining five were newly sequenced species in this study. These *Bauhinia* s.s. species were collected from Yunnan and Fujian provinces in China, representing medicinal species from Asia and Africa. Healthy and tender leaves were obtained. Total genomic DNA was extracted from the leaves using the DNA Quick Plant System (TIANGEN BIOTECH Co., Ltd., Beijing, China). The purity and integrity of the extracted DNA were assessed using AMPure XP system (Beckman Coulter, Beverly, MA, USA). After the DNA samples were qualified, the Agilent 5400 (Agilent Technologies Inc., Santa Clara, CA, USA) was used to detect the insert fragments of the library, and the expected insert fragments were accurately quantified using the Q-PCR method to ensure the quality of the library. Sequencing of the obtained library was conducted on the NovaSeq 6000 platform (Illumina, San Diego, CA, USA), generating 150 bp paired-end reads. The raw sequencing data were filtered using Fastp v0.23.4 [86] to remove low-quality reads, adapter sequences, and sequences containing more than 10% N bases, resulting in clean reads ranging from 5.96 to 8.23 GB in size.

### 4.2. Assembly and Annotation of the Chloroplast Genomes

The filtered clean reads were assembled using the Getorganelle v1.7.7 software [87], and the circularization of the cp genomes for five species was manually addressed using the Bandage v.0.8.1 tool [88]. *Bauhinia brachycarpa* (MF135595) [56], a congeneric species with high-quality annotation, was selected as a reference. The preliminary annotations of the obtained cp genomes were conducted using the CPGAVAS2 online platform (http://47.96.249.172:16019/analyzer/annotate (accessed on 20 November 2024)), followed by further manual correction, primarily involving the gene names, start codons, and stop codons. Finally, the annotated sequence files were uploaded to the CPGView website (http://www.1kmpg.cn/cpgview (accessed on 23 November 2024)) to generate physical maps of the complete cp genomes.

### 4.3. Codon Usage Bias Analysis

The common CDS from the five *Bauhinia* s.s. species assembled here were extracted, with sequences that were duplicate, incomplete, or shorter than 300 bp being excluded. CDS were selected with start codons of ATG and stop codons of TAA, TAG, and TGA. The filtered CDS sequences were concatenated and the CodonW v1.4.2 software [89] was used to calculate the RSCU value for each codon.

ENC reflects the degree of non-equilibrium usage of synonymous codons within a codon and is valuable for evaluating the overall codon preference of a gene. The Cusp tool from the Emboss v6.6.0 software [90] was used to calculate the guanine or cytosine (GC) content at the first (GC1), second (GC2), and third (GC3) positions of all codons in each CDS, and the Chips tool was employed to compute the ENC value. Scatter plots were then drawn with GC3 on the x-axis and ENC on the y-axis. Genes located above the standard curve indicate that their codon preference is solely influenced by base mutations, while those below the curve suggest that codon preference is also affected by natural selection and other factors.

PR2 analysis enables the detection of whether mutations at the third position of each codon are balanced. We selected amino acids that contained four degenerate synonymous codons and calculated the content of the four bases at the third codon position separately. The PR2 plot was then constructed, with G3/(G3 + C3)|4 as the x-axis and A3/(A3 + T3)|4 as the y-axis. The degree and direction of base bias were judged by the distance of each point from the center point. In the absence of base mutations or biases, the base content should be A = T and C = G.

The neutral plot analysis examines the correlation between the GC content at the third position (GC3) and the composition of bases at the first and second positions (GC1 and GC2) to investigate the influence on codon preference. A scatter plot was drawn with the GC3 of each gene as the abscissa and GC12 as the ordinate. If there is a significant correlation between the GC content at the third position and the bases at the first and second positions, it suggests that the codon preference of the gene is greatly influenced by base mutations, rather than by natural selection. Conversely, if there is no significance and the regression coefficient is close to 0, it indicates that the composition of bases at the first and second positions differs from that at the third position, and the GC content of the genome is conserved. This leads to the inference that the codon preference of the gene is influenced not only by base mutations but also by natural selection and other factors.

### 4.4. Comparative Analysis of the Chloroplast Genomes

Using the five cp genomes assembled in this study, with *B. brachycarpa* var. *microphylla* as the reference, a sequence conservation analysis of the cp genomes of *Bauhinia* s.s. was performed using the Shuffle-LAGAN model on the mVISTA online website (https://genome.lbl.gov/vista/mvista/submit.shtml (accessed on 23 November 2024)). The CPJSdraw v1.0.0 software [91] was used to visualize the expansion and contraction of the IR boundaries within the cp genomes of *Bauhinia* s.s. species.

### 4.5. Analysis of Repeat Sequences

The dispersed repeats in the cp genomes from *Bauhinia* s.s. were identified using the Reputer online tool (https://bibiserv.cebitec.uni-bielefeld.de/reputer (accessed on 23 November 2024)), including four types of repeats: forward (F), reverse (R), palindromic (P), and complementary (C) repeats. The hamming distance was set to 3, with a maximum computation of 5000 repeats and a minimum repeat length of 30. Additionally, simple sequence repeats were detected using the MISA v2.1 tool [92], with thresholds set for mononucleotides: 10; dinucleotides: 5; trinucleotides: 4; and tetranucleotides, pentanucleotides, and hexanucleotides all set to 3.

### 4.6. Hypervariable Region Identification and Selective Pressure Analysis

From the five cp genomes assembled here, we isolated the common CDS and IGS sequences. Utilizing DnaSP v.5.10 software [93], we detected nucleotide polymorphism (Pi) with a specified window length of 800 bp and a step size of 200 bp. The results obtained were then visualized through the ChiPlot website (https://www.chiplot.online/ (accessed on 28 November 2024)). Understanding selective pressure offers insights into the evolutionary forces shaping genes or genomes within species. For this analysis, the nonsynonymous substitution rate (Ka) and synonymous substitution rate (Ks) are pivotal parameters. Ka measures mutations in CDS that result in amino acid changes, whereas Ks reflects mutations that do not alter amino acids. Both parameters are essential for selective pressure analysis, and the Ka/Ks ratio serves as a frequently used indicator to evaluate the selective pressure acting on genes.

Based on the YN model, we calculated the Ka/Ks ratio using KaKs_Calculator v3.0 [94]. We tested the hypothesis of purifying selection in genes with Ka/Ks < 1 and positive selection in genes with Ka/Ks > 1. The CODEML tool in PAML v4.9 software [95] was used for branch-site analysis to detect positively selected sites on the foreground branch. Two models were selected: a null branch-site model (model = 2, NSsites = 2, fix_omega = 1, omega = 1) and an alternative branch-site model (model = 2, NSsites = 2, fix_omega = 0, omega = 2) [96]. The Likelihood Ratio Test (LRT) and Bayes Empirical Bayes (BEB) analysis were used to compare the two models and assess the likelihood of positive selection, with the chi-square tool in PAML v4.9 [95] used to calculate *p*-values. When the LRT value for a site was less than 0.05 and the BEB value was greater than 0.95, the site was considered to be under positive selection. Finally, the secondary structure of proteins containing positively selected sites was predicted using the PSIPRED website (http://bioinf.cs.ucl.ac.uk/psipred/ (accessed on 24 November 2024)) and the SWISS-MODEL online tool (https://swissmodel.expasy.org/ (accessed on 24 November 2024)).

### 4.7. Phylogenetic Analysis and Divergence Time Estimation

To determine the phylogenetic positions of the five obtained species, this study conducted a phylogenetic analysis using the complete cp genome sequences of twenty Cercidoideae species, including seven *Bauhinia* s.s. species, with *Cercis canadensis* (KF856619) [97] and *Cercis chinensis* (MZ128523) [98] serving as outgroups. The cp genome sequences were divided into three regions, namely, the LSC region, the SSC region, and the IR region. Subsequently, these regions were aligned using Mafft v7.520 software [99], followed by filtering of poorly aligned regions using trimAl v1.4 software [100] and then concatenation. Both the maximum likelihood (ML) and the Bayesian inference (BI) methods were employed to construct the phylogenetic tree. The PartitionFinder v2.1.1 tool was utilized to determine the optimal tree-building model, with the result indicating that the best-fit model for all three regions was the GTR + I + G model. The ML tree was then constructed using the IQ-Tree v2.0.3 software [101], with bootstrap values set to 1000. When constructing the BI tree using Mrbayes v3.2.7a software [102], the MCMC algorithm was run for 2,000,000 generations, with a sampling frequency of once every 1000 generations. The burn-in was set at 25%, and trees generated before convergence were discarded to obtain trees with posterior probabilities. Finally, the ChiPlot website (https://www.chiplot.online/ (accessed on 28 November 2024)) was used for the visualization of the phylogenetic tree.

With the basal node of Cercidoideae set at 66.0 Mya, and with fossil calibration points assigned to *Bauhinia* s.s. and *Cercis* at 56.0 Mya and 34.0 Mya, respectively, the former representing the earliest fossil record of *Bauhinia* s.s. [32]. The divergence times of species were estimated using the baseml and mcmctree tools from PAML v4.9 [95].

### 4.8. Sequence-Based Species Delimitation

The Assemble Species by ASAP method was employed to assess species delimitation. ASAP utilizes a hierarchical clustering algorithm and confirms the optimal species delimitation through “asap-scores”. The sequence files were uploaded to the ASAP website (https://bioinfo.mnhn.fr/abi/public/asap/asapweb.html (accessed on 28 December 2024)), and the Simple Distance (p-distances) model was selected to estimate genetic distances, with all other parameters remaining at their default settings. We used the Fastachar v0.2.4 software to calculate the MDC of each MOTU [103].

## 5. Conclusions

In this study, chloroplast genomes from five species of *Bauhinia* s.s. distributed across Africa and Asia were assembled, annotated, and subsequently compared and analyzed. The results revealed certain differences between African and Asian species in their cp genomes. (1) In terms of cp genome structure, *B. purpurea* distributed in the Indian Plate exhibited an expansion of approximately 300 bp in the IR region, while the IR regions of African species contracted by about 100–400 bp, with a slightly lower GC content than that of Asian species. The proportion of dispersed repeats in the IR regions of African species (44.68–49.15%) was relatively smaller compared to that of Asian species (69.23–71.62%), and the number of such sequences was also lower in African species. (2) Through SSR analysis, we identified six IGS regions with high Pi values, namely, *rpl33*-*rps18*, *trnP*-*psaJ*, *psbF*-*psbE*, and *ndhJ*-*ndhK* located in the LSC region, and *ndhI*-*ndhA* and *rpl32*-*trnL* located in the SSC region. These sites may be used for further research on the phylogenetic evolution. (3) In phylogenetic analysis, the two major lineages of *Bauhinia* s.l. received high support, with the African species *B. monandra* and *B. galpinii* clustering together and receiving strong support. (4) Branch-site analysis indicated that the *rps18* and *cemA* genes were under positive selection. We speculated that following the divergence of *Bauhinia* s.s. species in the late Paleocene (ca. 59.22 Mya), they underwent specific adaptive evolution in photosynthesis and cp function to adapt to the different growth environments in Asia and Africa. These findings not only highlight the genetic divergence between African and Asian Bauhinia species, but also provide valuable insights into their evolutionary history and potential adaptive strategies, laying a foundation for further research into the phylogenetic evolution within the genus.

## Data Availability

The chloroplast genome sequences supporting this study have been uploaded to GenBank (National Center for Biotechnology Information) with the accession number PQ826992-PQ826996. The BioProject ID is PRJNA1195732, with BioSample identifiers as SAMN45227226-SAMN45227230. And the corresponding SRA numbers are SRR31707633-SRR31707637.

## Supplementary Materials

The following supporting information can be downloaded at: https://www.mdpi.com/article/10.3390/ijms26010397/s1.
