# Supplementary material for: Comparative Analysis of Complete Chloroplast Genomes and Phylogenetic Relationships in Medicinally Important Pantropical Genus Bauhinia s.s. (Leguminosae) from Southern Africa and Eastern Asia
Source: Int J Mol Sci. 2025 Jan 5;26(1):397. doi: 10.3390/ijms26010397 (PMC11720137; doi:10.3390/ijms26010397)
Supplement: Supplementary file 1 [file ijms-26-00397-s001.zip › Caption of Supplementary Materials.pdf]

Table S1: Chloroplast genomic information for *Bauhinia* s.s. species;
Table S2: Functional annotation of chloroplast genes of *Bauhinia* s.s. species;
Table S3: The Relative Synonymous Codon Usage analysis of *Bauhinia* s.s. species;
Table S4: Neutrality plot analysis of *Bauhinia* s.s. species;
Table S5: Effective Number of Codons plot analysis of *Bauhinia* s.s. species;
Table S6: Parity Rule 2 plot analysis of *Bauhinia* s.s. species;
Table S7: Statistics of dispersed repeats from *Bauhinia* s.s. species;
Table S8: Distribution of dispersed repeats in different regions for *Bauhinia* s.s. species;
Table S9: Statistics of SSRs from Bauhinia s.s. species;
Table S10: Distribution of SSRs in functional regions from *Bauhinia* s.s. species;
Table S11: The Pi value of protein-coding genes and intergenic spacer regions from *Bauhinia* s.s. species;
Table S12. Numbers of MDCs within five MOTUs;
Figure S1: Comparison of the LSC, IR, and SSC boundary regions in the chloroplast genomes of *Bauhinia* s.s. species. JLB, JSB, JSA, and JLA represent the connections between LSC and IRb, IRb and SSC, SSC and IRa, and IRa and LSC, respectively. The distance between genes and the length of each region are marked in the figure with numbers;
Figure S2: Sequence similarity of *Bauhinia* s.s. species was analyzed using mVISTA. The chloroplast genome of *Bauhinia brachycarpa* var. *microphylla* was used as the reference genome. The horizontal axis represents the gene position of the reference sequence, while the vertical axis indicates the similarity ranging from 50% to 100%. Exons, t/rRNAs, and conserved noncoding sequences (CNS) are represented by different colors in the lower left corner.
