# Supplementary figures and images for: Comparative Analysis of Complete Chloroplast Genomes and Phylogenetic Relationships in Medicinally Important Pantropical Genus Bauhinia s.s. (Leguminosae) from Southern Africa and Eastern Asia
Source: Int J Mol Sci. 2025 Jan 5;26(1):397. doi: 10.3390/ijms26010397 (PMC11720137; doi:10.3390/ijms26010397)

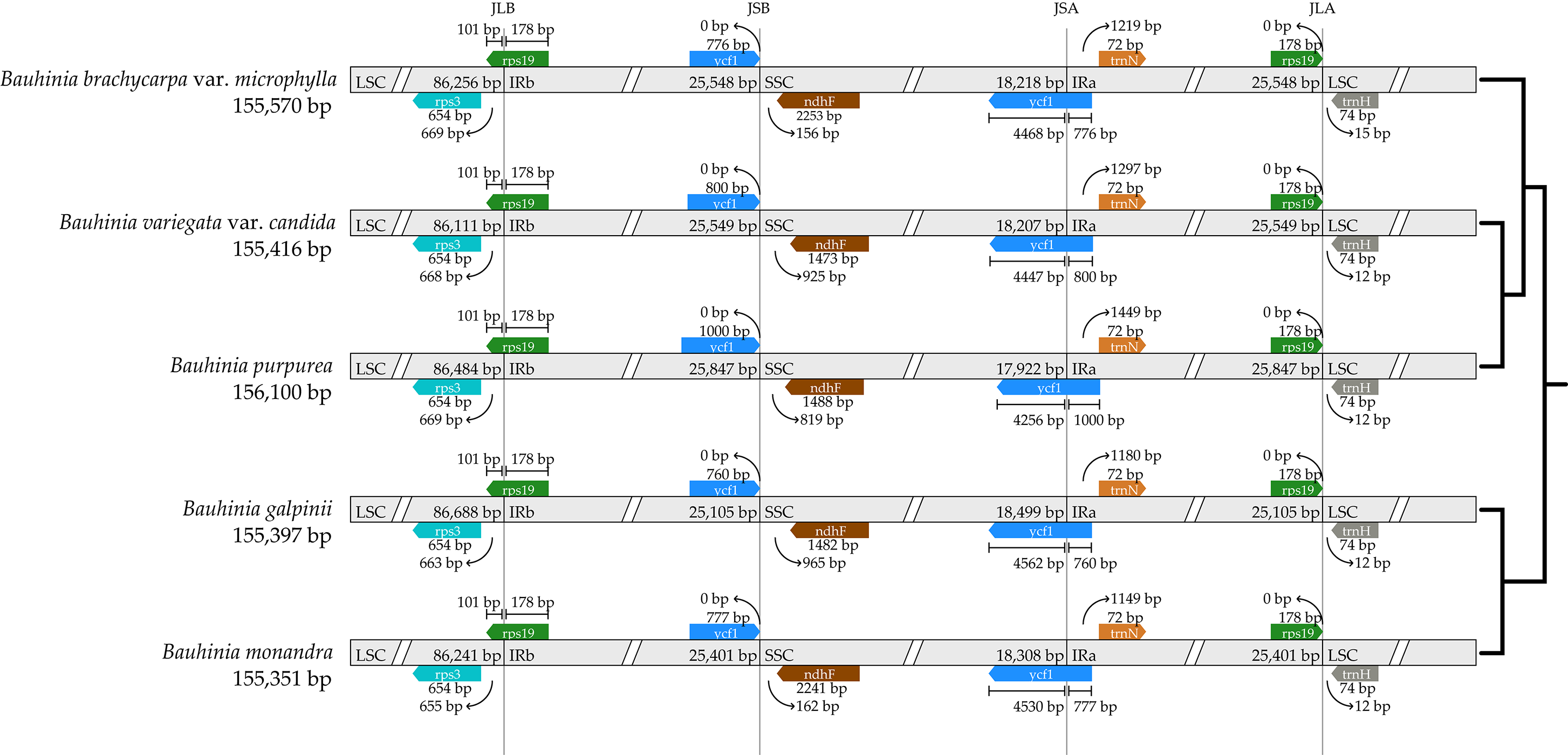

Supplement: Supplementary file 1 [file ijms-26-00397-s001.zip › Figure S1.tif]

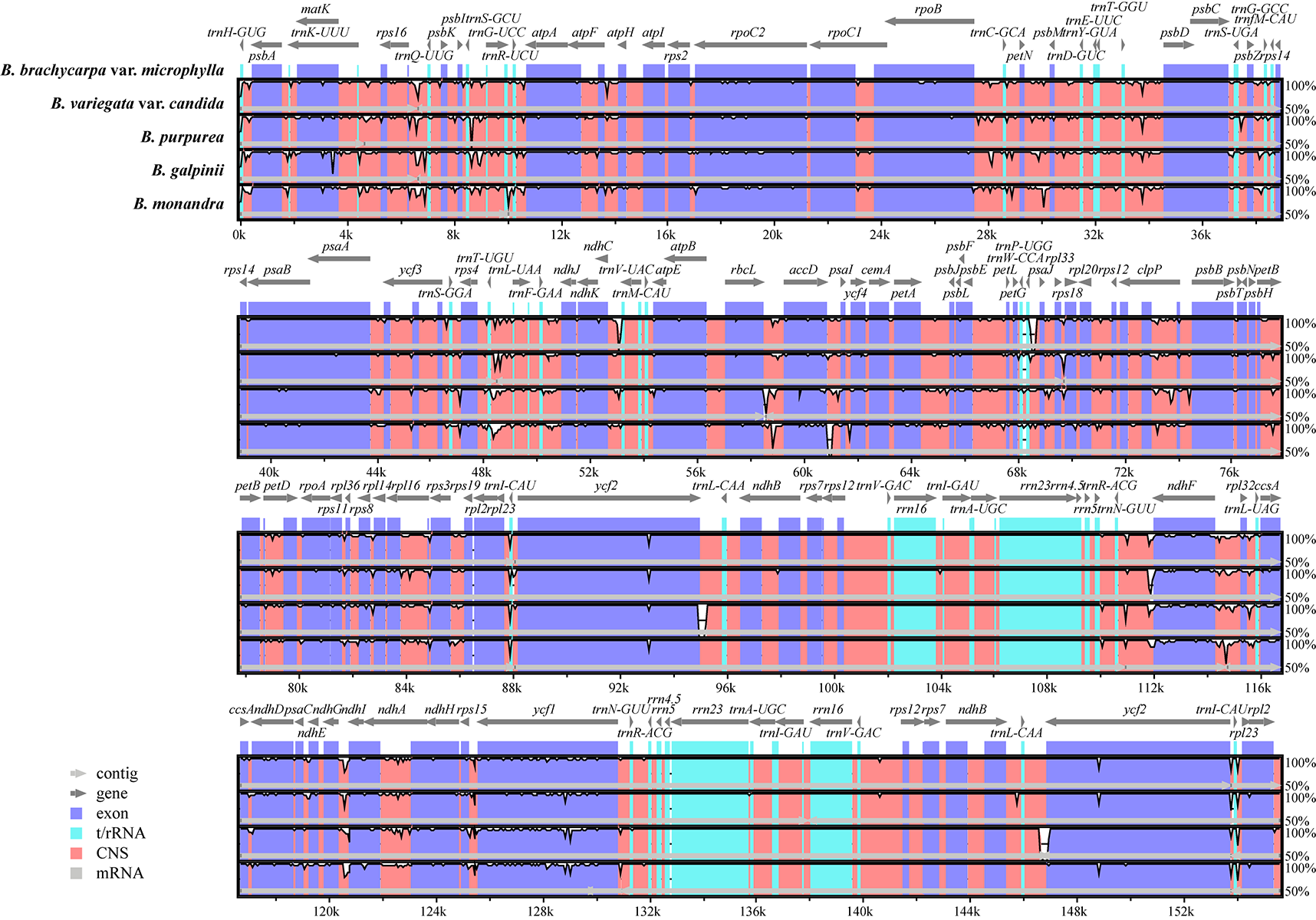

Supplement: Supplementary file 1 [file ijms-26-00397-s001.zip › Figure S2.tif]
